# Cephalometric evaluation of soft tissue changes after extraction of upper first premolars in class ΙΙ div 1 patients

**DOI:** 10.4317/jced.51474

**Published:** 2014-12-01

**Authors:** Gholamreza-Eslami Amirabadi, Marzieh Mirzaie, Somayyeh-Mehrabi Kushki, Pooya Olyaee

**Affiliations:** 1 Assistant professor, Shahed university of medical sciences; 2 Assistant professor, Urmia university of medical sciences

## Abstract

Introduction: Tooth extraction to provide sufficient space, or camouflage of underlying skeletal problems is quite common in orthodontics. The present study evaluated soft tissue changes after upper first premolars extraction in class ΙΙ div 1 patients. Material and Methods: 20 cases (15 females, 5 males), with a mean age of 17.8±2.9 years with class ΙΙ div1 malocclusion and normal vertical height, who needed upper first premolars extraction were selected. Pre- and post-treatment lateral cephalometric radiographs were digitized. Image analysis was conducted by View Box 3.1.1 software. Paired t-test was used for comparison of pre- and post-treatment results. Results: The relationship of upper and lower lip to E-line and B-line had significant reduction. Dental variables of U1-NA(mm), U1-NA(°), overjet and overbite showed statistically significant reduction. Interincisal angle had significant increase. There were no significant difference in lower incisor variables and skeletal variables like SN-GoGn and FMA. Conclusions: Extraction of upper first premolars in patients with class ΙΙ div 1 malocclusion resulted in normal position of lips as presented by Holdaway, Legan and Ricketts which play a role in aesthetic profile. However, the amount of lip retraction was different from patient to patient.

** Key words:**Soft tissue, fixed orthodontic treatment, class ΙΙ div 1, upper first premolar extraction.

## Introduction

One of the main concerns of orthodontic treatment is soft tissue changes after extraction of premolars and in the previous years, non extraction treatments and molar distalization have been popularized (1,2). Some researchers disagree with extraction of premolars because of consequences such as dish faces, flattening of the face and retraction of the lips (3-6). On the other hand, patients prefer more prominent lips these days. Although some researchers have reported that, patients’ preference has not affected orthodontic practice adversely (7,8). In general, an orthodontic fixed treatment consists of arch expansion in non-extraction treatments, and extractions in instances of severe crowding and protrusion (7,8). In cases with arch size/tooth size discrepancy of 5-9mm, non extraction and extraction treatment is possible and the treatment plan depends on the hard and soft tissue characteristics of the patient but if the discrepancy is 10 mm or more, extraction is almost always required. Four first premolars or perhaps upper first premolars are the extraction choice most times. Rarely, second premolar or molar extraction is satisfactory because it does not provide enough space in severely crowded patients (7). There are still ongoing debates on the effects of extraction on vertical height dimension, profile changes, jaw position, TMJ health and periodontal situation after treatment (7,9-15).

 The horizontal relationship of the lips has been proposed as an important characteristic in esthetics (16). Upper lip length increases during orthodontic treatment. Part of it is due to growth changes and the remaining is due to the bite alterations during treatment (17). E-line or aesthetic plane was introduced by Ricketts to evaluate the position of lips (17). Other planes such as S-line, B-line, H-line, … also were introduced to assess soft tissue alterations (17,18). There are different studies with controversial results on evaluating soft tissue after orthodontic treatments. Assuncao *et al.* reported that the upper lip length didn’t show significant changes due to retraction of incisors in adult patients (19). Bishara and Jacobson in a similar research found that, orthodontic treatment either by extraction or not, improve soft tissue profile of the patients (20). Lai *et al.* showed that soft tissue’s response was not predictable and so did Zarringhalam and Arash (21,22). Conley also found this result for his patients treated by extraction of upper premolars (2). Akyalcin and Hazar reported that, extraction for orthodontic treatment retruded the lips but non extraction treatments didn’t affect the profile too much (23). Tadiac *et al.* declared that by extraction of upper first premolars, nasolabial angle, upper and lower lip sulcus depth and position of upper incisors changed proportional to previous soft tissue characteristics and pre treatment incisor position and all of them relates to the ANB angle alterations (24). According to controversial results of these studies, this trial was done to evaluate soft tissue alterations after extraction of upper premolars in class ΙΙ div 1 patients.

## Material and Methods

In this cross-sectional clinical study, 15 females and 5 males with the following criteria were included: (1) 15 years or older [mean age: 17.8±2.9] (2) patients with class ΙΙ div 1 malocclusion without any missing except the third molars (3) normal vertical growth [20<FMA<29] (4) overjet>=4mm (5) upper first premolars were extracted for orthodontic treatment (6) pre- and post treatment lateral cephalometric radiographs with good properties were available (7) treatment was done by 0.018 standard edgewise system (8) no experience of using extra oral or functional appliances (9) orthosurgery was not included in the treatment plan. To reduce growth effect on the results, individuals less than 15 years were not chosen ( Table 1- Table 3). In this study, six skeletal variables, 18 soft tissue variables and 14 dental variables were evaluated and the results were declared in three groups: first, pa-tients with 4mm or less crowding [0<crowding<=4mm], second group composed of patients with more than 4 mm crowding [4<crowding<=6mm] and the third group composed of all the patients of first and second groups. Lateral cephalometric radiographs were digitized by canon 100 camera. Image analysis was conducted by view box 3.1.1 software. The data was analyzed by SPSS statistical software through paired t-test with confidence of 95% [ α=0.05]. Since the confidence base was supposed 95%, so if p-value was more than 0.05, null hypothesis was not rejected which shows no difference, and if *p*-value was less than 0.05, so significant difference was between the results statically.

**Table 1:**
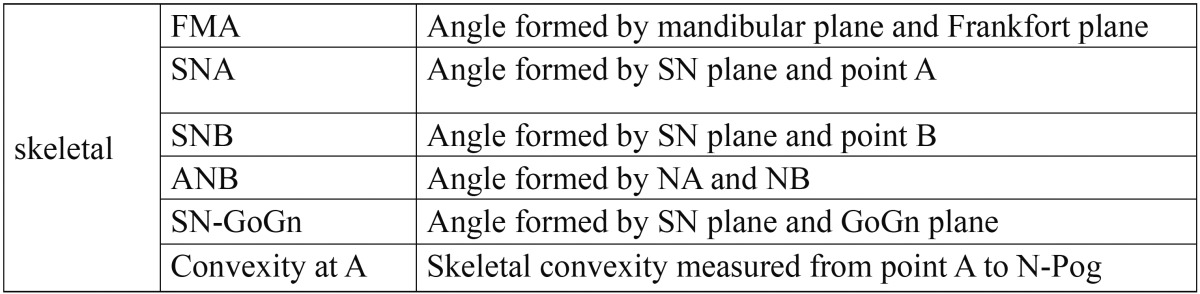
Skeletal variables.

**Table 2:**
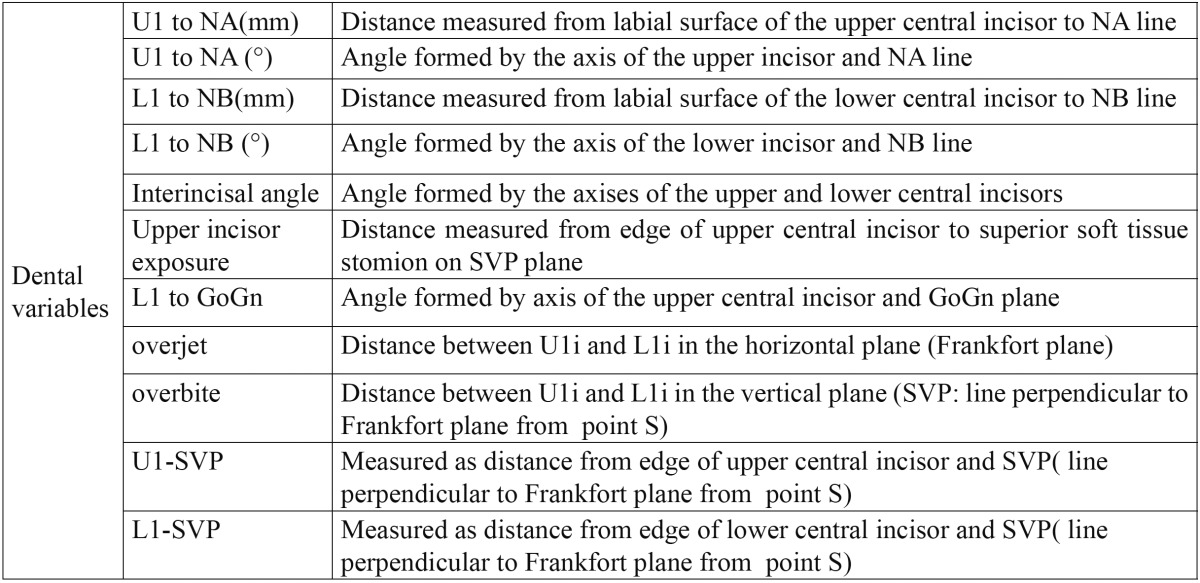
Dental variables.

**Table 3:**
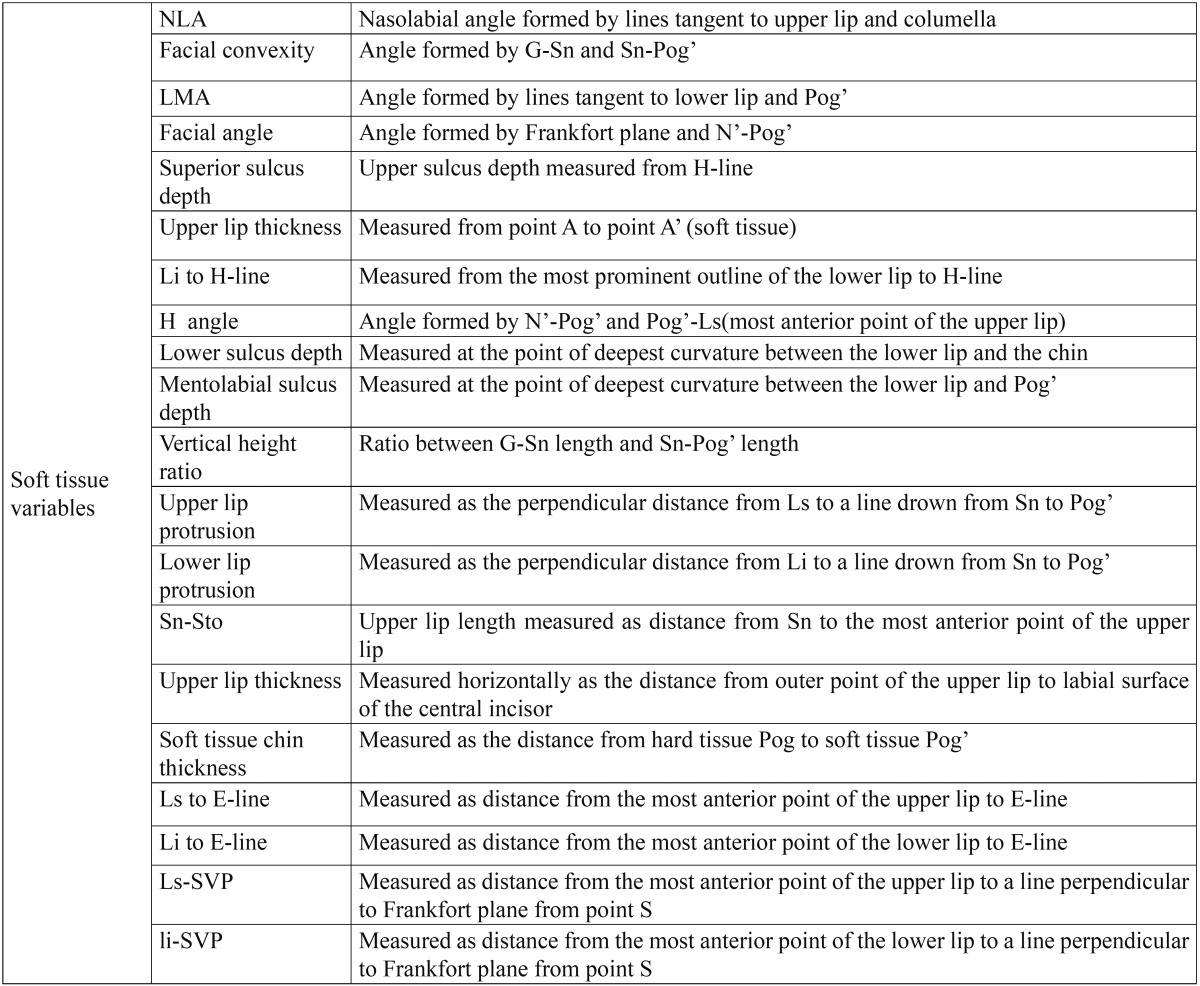
Soft tissue variables.

## Results

As told above, results of this study are discussed in three groups.

Soft tissue variables: In all groups, upper lip protrusion, Ls [upper lip] to E-line, Ls to SVP, superior sulcus depth and H angle had significant reduction. Li [lower lip] to E-line, lower lip protrusion and facial convexity decreased significantly in all groups except for lower lip protrusion and facial convexity in the first group which decreased but not significantly. Upper lip protrusion, nasolabial angle and labiomental angle increased significantly in all groups. Lower lip to H-line and soft tissue facial angle decreased in all groups but not statistically significant. Mentolabial sulcus depth and inferior sulcus to H-line slightly decreased in the first and third group and increased slightly in the second group. Sn-Sto increased in all groups but it was not significant. Soft tissue thickness increased insignificantly in the first and second groups and showed significant increase in the third group. Vertical height ratio [G-Sn/Sn-Pog’] didn’t change significantly ( Table 4).

**Table 4:**
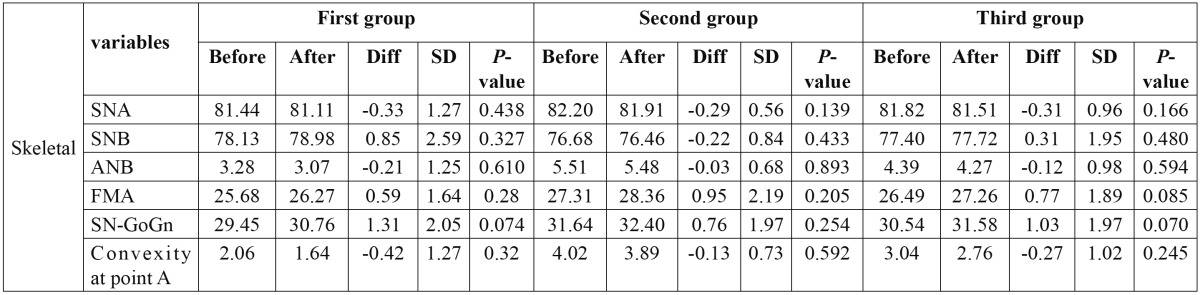
Data gained for skeletal variables, before treatment, after treatment, difference between them, SD and *p*-value.

Skeletal variables didn’t change significantly too. SNA, ANB and convexity at point A decreased and SNB and FMA increased but not significantly ( Table 5).

**Table 5:**
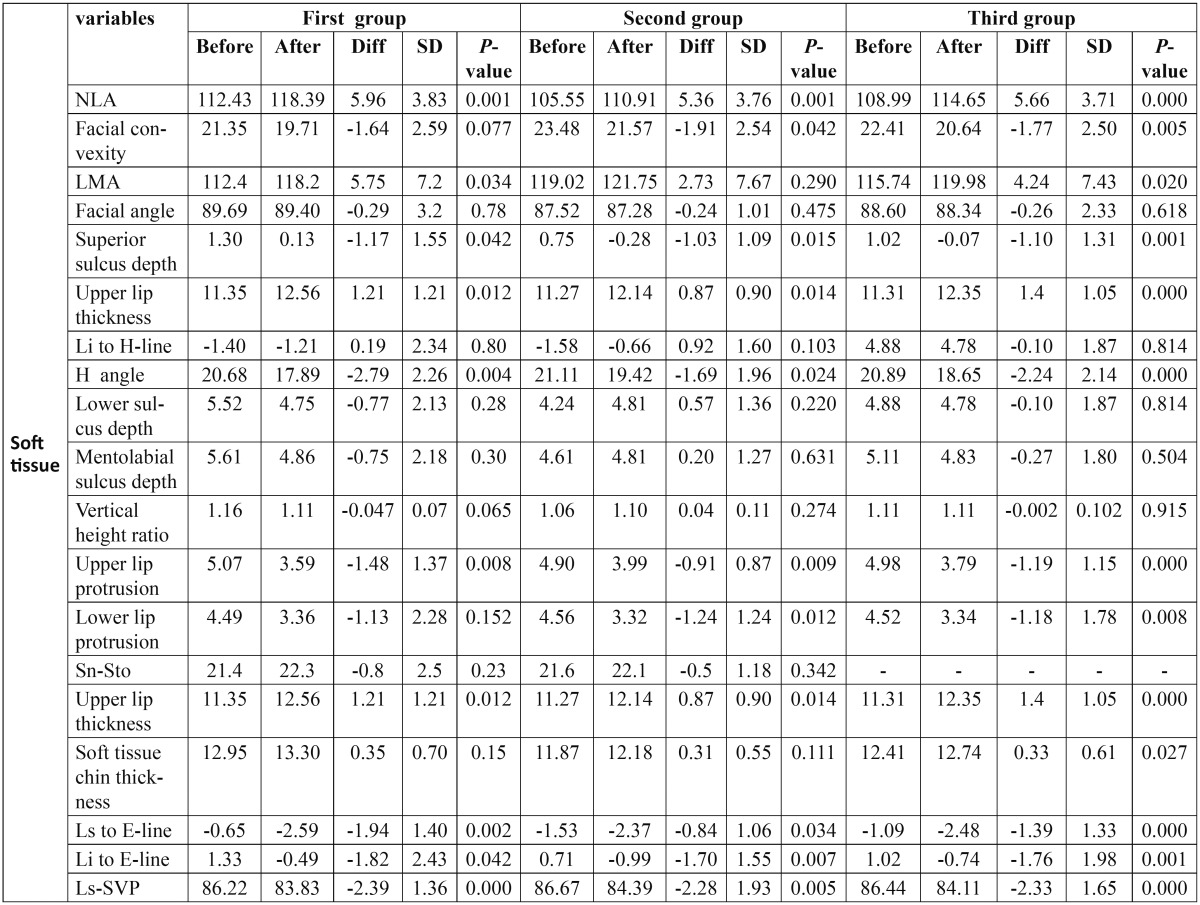
Data gained for soft tissue variables.

Dental variables: L1 to GoGn and L1 to NB increased insignificantly. U1 to NA[°], U1 to NA[mm], U1 to SVP, overjet and overbite decreased significantly. U1 exposure didn’t change in the first group but decreased in the second and third group which wasn’t significant. Interincisal angle increased in the first group and decreased in the second and third groups but not significantly ( Table 6).

**Table 6:**
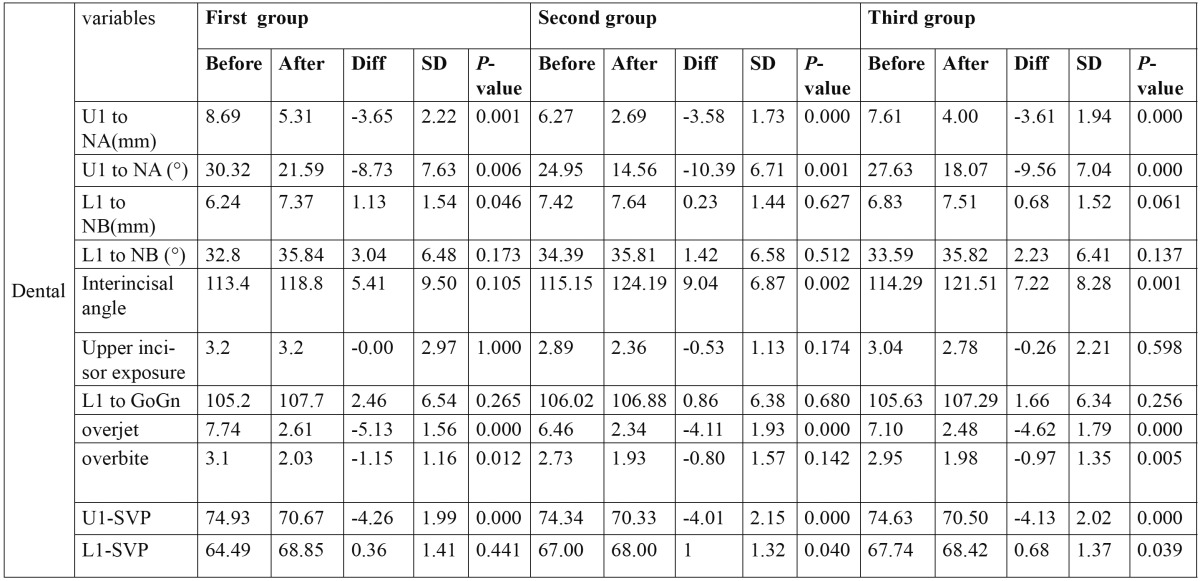
Data gained for dental variables.

## Discussion

In this study, all the patients were treated by camouflage therapy in which dental alterations mask the improper underlying skeletal relationship. Premolar extraction reduced protrusion of the upper incisors, and subsequently the prominence of the upper lip was reduced.

Growth may increase vertical dimension and therefore loss of vertical height dimension due to treatment by extraction of premolars can be masked with growth changes (23,24). It has been shown that extraction of the upper premolars do not have sensible effect on reduction of the vertical height dimension (21,25,26,27). In the present study, vertical skeletal variables such as FMA and GoGn to Sn didn’t change significantly. It can be concluded that vertical height dimension was controlled sufficiently although the patients were not at growing age.

In this study, SNA didn’t change significantly. A non significant reduction in SNA in mesiodivergent and hyper-divergent patients treated by headgear, has been documented (28). Cigar studied class ΙΙ patients with average age of 11 years and used cervical headgear during treatment, SNA decreased insignificantly and SNB didn’t show sensible alterations but after treatment SNB increased due to the remaining growth (29). Others have showed reduction in SNA (23,30). This may be due to extraction of lower premolars and class Ι dental relationship. In the present study headgear was not administered and SNA alterations were not significant.

ANB angle decreased insignificantly in this study as shown by other researchers (23,29,31). Taner showed significant reduction in the patients treated by headgear which was due to SNA alterations (27). As discussed above, protrusion of the upper incisors decreased in this study which had proper effect on profile, ANB and U1 to NA [°].

U1 to NA [mm] and U1 to SVP showed retrusion of the upper incisors as shown by other researchers (30-33). Kyung Kim didn’t show any significant change in this angle. It may be due to class Ι relationship of their patients and relieving the crowding by the extraction space without any effect on retrusion of the incisors (34).

L1 to NB [°] and L1 to NB [mm] didn’t show significant changes. It was one of the best results of this study, which depends on Class II elastic use, the amount of crowding in the lower rarch, and method of space provision in the lower arch. Therefore protrusion of the lower incisors may happen in class II patients with crowding in the lower arch treated non-extraction (31,32,34). Reduction in L1 to mandibular plane angle were due to growth potential of the patients (35). Class ΙΙ elastics and Begg appliance can increase L1 to NB [mm] significantly but cervical headgear may decrease it to a significant amount (33).

Downs found that for occlusal stability, interincisal angle should be 135.4° (36). Interincisal angle increased to 121° after treatment in our study. Aslihan has also showed increase in this angle after treatment (35). Ideal interincisal angle was 131° in Steiner norms (37).

Upper lip does not show steady and predictable response to retraction of upper incisors (2,38). However, our study showed, Ls to E-line, upper lip protrusion and Ls to SVP decreased significantly which showed retrusion of the upper lip to the normal position. The original lip competency and degree of lip separation at rest are among factors affecting lip response to extraction therapy.

Sn-Sto which shows the upper lip length, didn’t change. Park *et al.* found the same result (1). Lower lip to E-line and lower lip protrusion decreased in this study, although the lower incisors were protruded. The reason is that, the lower lip covers the upper incisors and retrusion of the upper incisors not only affects the upper lip but also retrudes the lower lip. Lai *et al.* reported that Li to E-line decreased significantly but it was due to the extraction of the lower premolars (22).

Lower lip to H-line slightly increased. This is because of more retrusion of the upper lip than the lower lip. Bloom and Jacobs reached the fact that the relationship between retrusion of the lower lip and retrusion of the upper incisors was more than retrusion of the lower incisors (39,40).

De Smit and Dermaut found 110° as the ideal nasolabial angle (41). In this study, this angle increased 5.44° and the mean amount for all the patients was 114.65°. Akyalcin and Hazar showed significant reduction in this angle in both extraction and non extraction groups of patients (23).

In a study of De Smit and Dermaut, deep geniolabial sulcus was preferred aesthetically than flat geniolabial angle (41). In this trial, mentolabial sulcus depth and inferior sulcus to H-line decreased but it was not significant. Kocadereli found that inferior sulcus to E-line didn’t change significantly either in extraction group or non extraction group (3).

Upper lip thickness increased to a significant amount about 1.4mm, which is in agreement with other researchers (22,38,42-44). Holdaway declares that strained lips reach their natural tonus at first by retraction of the incisors and then follow the retraction of the lip by the proportion of 1:1 (44). In this study, it is concluded that extraction of upper first premolars in patients with class ΙΙ div 1 malocclusion results in normal position of lips as presented by Holdaway, Legan and Ricketts which play a role in aesthetic profile.

One of the shortcomings of the present study is that we have not considered the amount of lower arch crowding [although all the cases were non-extraction in the lower arch] which may affect our results, because of the effects of lower arch expansion on the facial and dental variables, it is suggested that future studies categorize the cases considering the amount of lower arch crowding.

## References

[1] Parks S, Kudlick EM (1989). Vertical dimensional change of the lips in the North American Black patient after four first premolar extractions. Am J Orthod Dentofacial Orthop.

[2] Conley R, Jernigan C (2006). Soft tissue change after upper premolar extraction in class ΙΙ camouflage therapy. Angle Orthod.

[3] Kocadereli I (2002). Changes in soft tissue profile after orthodontic treatment with and without extraction. Am J Orthod Dentofacial Orthop.

[4] Nelson RT, Russell DM (1986). Facial soft tissue profile changes in the North American Black with four first bicuspid extractions. J Md State Dental Assoc.

[5] Kusnoto J, Kusnoto H (2007). The effect anterior tooth retraction on lip position of orthodontically treated adult Indonesians. Am J Orthod Dentofacial Orthop.

[6] Leonardi R, Annunziata A, Licciardello V, Barbato E (2010). Soft tissue changes following the extraction of premolars in nongrowing patients with bimaxillary protrusion. A systematic review. Angle Orthod.

[7] Stephens CK, Boley JC, Behrents RG, Alexander RG, Buschang PH (2005). Long term profile changes in extraction and nonextraction patients. Am J Orthod Dentofacial Orthop.

[8] Brandet S, Safirestein GR (1975). Different extraction for different malocclusion. Am J Orthod.

[9] Weintrau JA, Vig PS, Brown C, Kowalski CJ (1989). The prevalence of orthodontic extraction. Am J Orthod Dentofacial Orthop.

[10] Tweed CH (1944). Indications for extraction of teeth in orthodontic procedure. Am J Orthod.

[11] Saatci P, Yukay F (1997). The effect of premolar extraction on tooth size discrepancy. Am J Orthod Dentofacial Orthop.

[12] Hahn G (1975). Extraction Panel Orthodontics: It's objective, past and present. Am J Orthod.

[13] De Castro N (1974). Second premolar extraction in clinical practice. Am J Orthod.

[14] Begg PR (1954). Stone age man's dentition. Am J Orthod.

[15] Orton HS, Slattery DA, Orton S (1992). The treatment of severe 'gummy' Class II division 1 malocclusion using the maxillary intrusion splint. Eur J Orthod.

[16] Hamdan AM, Rock WP (2001). Cephalometric norms in an Arabic population. Journal of Orthodontics.

[17] Anderson G, Fields HW, Beck M, Chacon G, Vig KW (2006). Development of cephalometric norms using a unified facial and dental approach. The Angle Orthodontist.

[18] Valentim ZL, Capelli Júnior J, Almeida MA, Bailey LJ (1994). Incisor retraction and profile changes in adult patients. Int J Adult Orthodon Orthognath Surg.

[19] Bishara SE, Jakobsen JR (1997). Profile changes in patients treated with and without extraction: Assessment by lay people. Am J Orthod Dentofacial Orthop.

[20] Zarringalam M, Arash V (2010). Labial changes following extraction of first premolars for orthodontic treatment in patients with malocclusion class ΙΙ div 1. Mashhad J.

[21] Lai J, Ghosh J, Nanda RS (2000). Effects of orthodontic therapy on the facial profile in long and short vertical facial patterns. Am J Orthod Dentofacial Orthop.

[22] Hazar S, Akyalcin S, Boyacioglu H (2003). Soft tissue profile changes in Anatolian Turkish girls and boys following orthodontic treatment with and without extraction. Turk J Med Sci.

[23] Tadic N, Woods MG (2007). Incisal and soft tissue change effect of maxillary premolar extraction in class ΙΙ treatment. Angle Orthod.

[24] Staggers JA (1990). A comparison of results of second molar and first premolar extraction treatment. Am J Orthod Dentofacial Orthop.

[25] Witzing JW, Spahl TJ (1987). The clinical management of basic maxillofacial orthodontic appliances. Littleton, Massachusetts: PSG publishing.

[26] Wyatt WE (1987). Preventing adverse effects on the TMJ through orthodontic treatment. Am J Orthod Dentofacial Orthop.

[27] Taner-Sarisoy L, Darendeliler N (1999). The influence of extraction orthodontic treatment on craniofacial sutures: Evaluation according to two different factors. Am J Orthod Dentofacial Orthop.

[28] Heravi F, Sahafian SH (2004). Facial vertical changes among patients treated by edgewise orthodontic technique along with tooth extraction. Journal of Dentistry. Tehran University of Medical Sciences.

[29] Ciger S, Aksu M, Germeç D (2005). Evaluation of post treatment changes in class ΙΙ div 1 patients after nonextraction orthodontic treatment. Am J Orthod Dentofacial Orthop.

[30] Ahn JG, Schneider BJ (2000). Cephalometric appraisal of post treatment vertical change in adult orthodontic patients. Am J Orthod Dentofacial Orthop.

[31] Hagler BL, Lupini J, Johnston LE Jr (1998). Long term comparison of extraction and nonextraction alternatives in mached samples of African American patients. Am J Orthod Dentofacial Orthop.

[32] Nelson B, Hägg U, Hansen K, Bendeus M (2007). A long term follow up study of class ΙΙ elastics or fixed functional appliances. Am J Orthod Dentofacial Orthop.

[33] Kim TK, Kim JT, Mah J, Yang WS, Baek SH (2005). First and second premolar extraction effects on facial vertical dimensions. Angle Orthod.

[34] Erdinc AE, Nanda RS, Dandajena TC (2007). Profile changes of patients treated with and without premolar extractions. Am J Orthod Dentofacial Orthop.

[35] Downs WB (1956). Analysis of the dentofacial profile. Angle Orthod.

[36] Steiner CC (1959). Cephalometrics in clinical practice. Am J Orthod.

[37] Wisth PJ (1974). Soft tissue response to upper incisor retraction in boys. Br J Orthod.

[38] Bloom LA (1961). Perioral profile changes in orthodontic treatment. Am J Orthod.

[39] Jacobs JD (1978). Vertical lip changes from maxillary incisor retraction. Am J Orthod.

[40] De Smit A, Dermaut L (1984). Soft tissue profile preference. Am J Orthod.

[41] Ricketts RM (1960). Cephalometric synthesis. Am J Orthod.

[42] Anderson JP, Joondeph DR, Turpin DL (1973). A cephalometric study of profile changes in orthodontically treated cases ten years out of retention. Angel Orthod.

[43] Roos N (1977). Soft tissue profile changes in class ΙΙ treatment. Am J Orthod.

[44] Holdaway RA (1983). A soft tissue cephalometric analysis and its use in orthodontic treatment planning. Part I. Am J Orthod.

